# MOODMIND: Artificial Intelligence for Major Depressive Disorder Screening in Tuberculosis Patients

**DOI:** 10.12688/f1000research.168964.1

**Published:** 2025-10-13

**Authors:** Erlina Wijayanti, Ammar Abror, Ummi Azizah Rachmawati, Citra Fitri Agustina, Helwiah Umniyati, Diana Batara Munti, Exir Najib Rahmat, Athoillah Ahkam Diansyah

**Affiliations:** 1 Family Medicine Primary Care Study Program, Faculty of Medicine, Yarsi University, Central Jakarta, Jakarta, Indonesia; 2 Faculty of Information Technology, Yarsi University, Jakarta, Indonesia; 3 Department of Psychiatry, Faculty of Medicine, Yarsi University, Jakarta, Indonesia; 4 Faculty of Dentistry, YARSI University, Jakarta, Indonesia; 5 Faculty of Medicine, Yarsi University, Jakarta, Indonesia

**Keywords:** Artificial intelligence, depression, detection, tuberculosis, Natural Language Processing

## Abstract

**Background:**

Major Depressive Disorder (MDD) can occur in patients with tuberculosis. The purpose of this research was to develop an early detection system for MDD and conduct an accuracy test.

**Methods:**

The MOODMIND application uses Natural Language Processing (NLP) with sentiment analysis techniques. MOODMIND offers both speech and text options and is available in Indonesian/English. The screening results were compared with those of the doctor’s autoanamnesis test. Single blinding is used so that doctors are unaware of the application test.

**Results:**

The app asks open- and closed-ended questions for MDD identification based on the DSM-5. The test results were divided into non-depressive (none or at-risk) and suspected depression groups. MOODMIND showed 67% sensitivity and 100% specificity.

**Conclusions:**

Ease is advantageous because the steps are simple. MOODMIND has sufficient accuracy, but it can be improved by adding words related to depression in the lexicon adjustment.

## 1. Introduction

Tuberculosis (TB) is a chronic infectious disease that requires at least 6 months of therapy. Psychiatric conditions are important because patients with TB can experience social stigma, worries about their illness, or difficulties during treatment. Depression has a strong effect on negative outcomes. ^1^ Individuals who undergo treatment with second- and third-line medications are at a greater risk of stigma and depression. ^2^

Depression also affects the immune system by lowering CD3, CD4, C8, and lymphocyte. ^3^ Low serum anti-inflammatory cytokine levels are observed in patients with Major Depressive Disorder (MDD)-TB. Recognition of MDD in patients with TB will be more appropriate for diagnosis, treatment, and prognosis. ^4^

The prevalence of major depression is 322 million worldwide ^5^ and some patients do not seek help. Major depression has the potential to lead to suicide. Questionnaires and screening tools have been developed, but most use closed-ended questions, such as the Mental Health Screening Tool for Depressive Disorders (MHS:D). ^6^

Natural Language Processing (NLP) is an artificial intelligence capable of analyzing and interpreting words. ^7^ NLP can be used remotely for the real-time detection of depression. Studies have built systems with NLP to analyze the signs of depression based on comments on social media, such as mental health. The researchers compared mental health with the PHQ-9 to determine the accuracy of the system. ^8^

The NLP techniques used include sentiment analysis, linguistic markers, word embedding, convolutional neural networks, recurrent neural networks, and large language models. Sentiment analysis examines the tone of emotions in a text, referring to depression if a negative language is identified. ^9^

Based on the above description, a web-based application was built to screen for MDD using sentiment analysis. The software provides an alternative with open-ended questions on the two key symptoms for the diagnosis of major depression in both Indonesian and English. Through early detection, it is hoped that depression can be treated immediately and that this will increase the chances of successful treatment.

## 2. Methods

**A. MOODMIND development**

The project is part of an effort to examine tuberculosis patients holistically by developing AI-based tools for detecting MDD.

**1. Ethical considerations**

The ethics committee of YARSI University reviewed the ethical clearance number 114/KEP-UY/EA.20/III/2025.

**2. Implementation**

**Figure 1.**
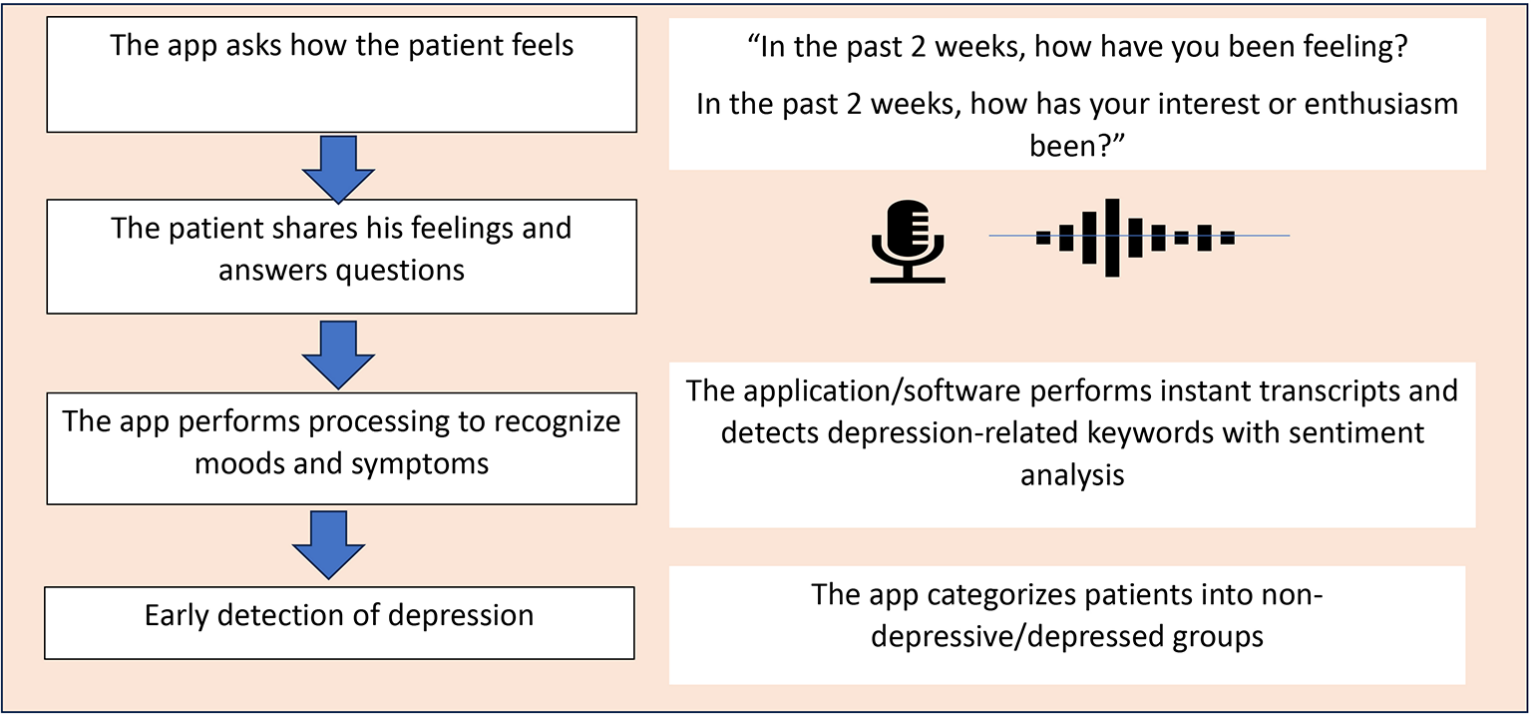
MOODMIND application concept for Major Depressive Disorder (MDD) screening.

**Figure 2.**
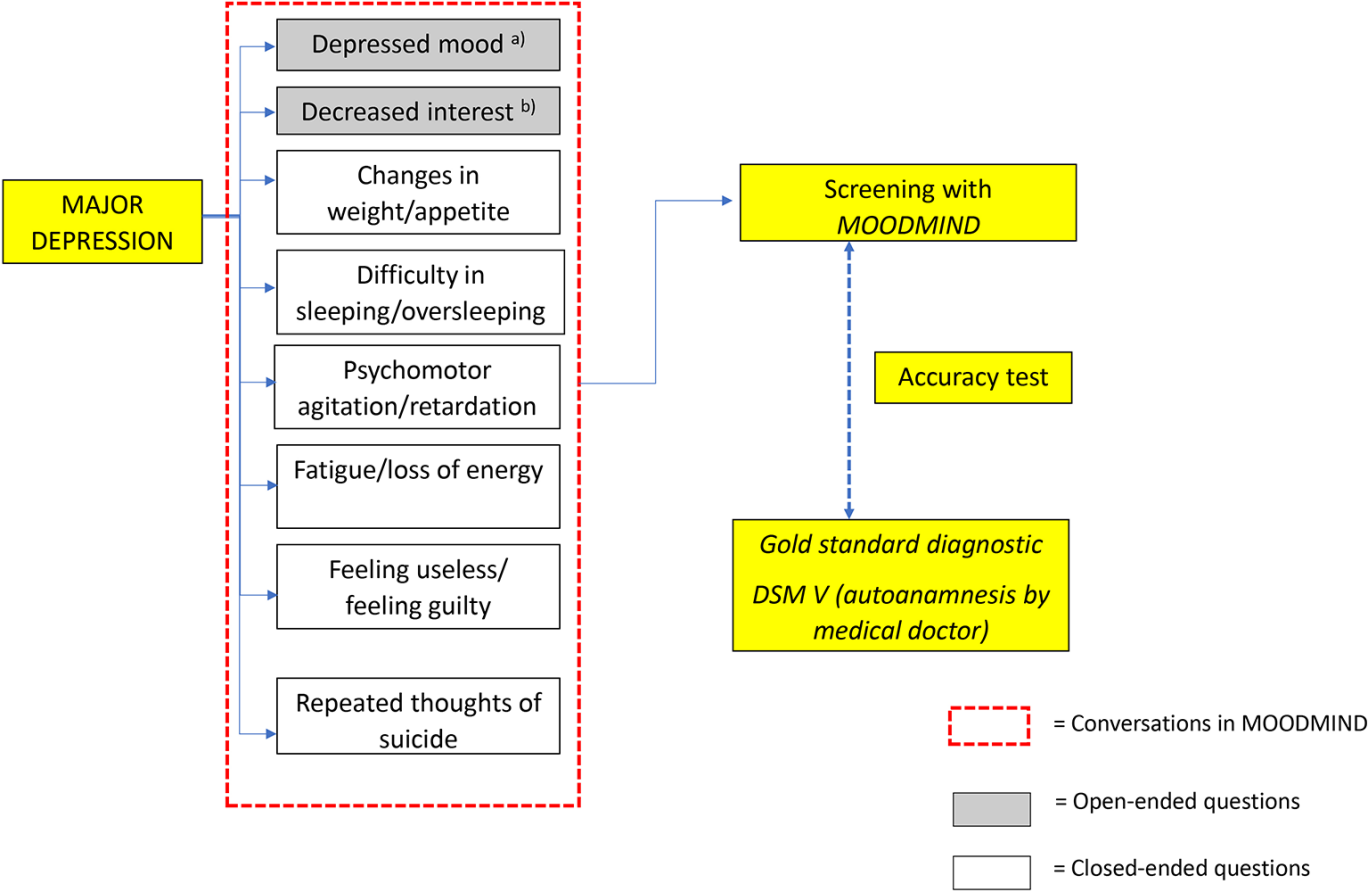
Conceptual framework for MOODMIND application development.

MDD is diagnosed if it meets the criteria of five or more symptoms (there is at least one symptom point a or b) for at least two weeks. ^10^ Figures 1 and 2 illustrate the concept of MOODMIND, respectively.

## 3. Operation

The software can be accessed via the following link: https://moodmind-two.vercel.app/.

### 3.1 Technologies

MOODMIND used Next.js for Frontend Framework, Tailwind CSS, and Web Speech API for Speech Recognition. The Programming Languages are TypeScript and JavaScript.

### 3.2 Main components

VoiceChat.tsx manages the voice input, transcripts, and conversation flow control. UseSpeech.ts for customizing hooks to control speech recognition status. The scripts provide questions and response scripts.

### 3.3 Depression detection methodology

The detection approach was based on several text-based indicators derived from voice transcription, namely, language patterns and depression-related keywords.

### 3.4 User experience flow

Users open the web-based application and answer system questions using voice or text. The system processes the transcription using sentiment analysis. The results of the analysis are displayed in visual and narrative forms.

### 3.5 Adaptation for tuberculosis

MOODMIND was adapted with a custom sentiment dictionary, focusing on common terms in Bahasa Indonesia that were reported by patients with TB when experiencing emotional distress.

### 3.6 Implementation details in sentiment analysis integration

As part of its natural language processing features, this system is equipped with a sentiment analysis module to evaluate the emotions contained in voice recognition transcripts. Sentiment analysis aimed to identify the emotional orientation (positive, negative, or neutral) of a statement, which, in this context, was used to detect indications of mood and enthusiasm in patients. Sentiment analysis was performed using the sentiment library, an open-source JavaScript library that supports lexicon-based analysis.

**Lexicon adjustments for Indonesian**

By default, a sentiment library supports the English language. To support Indonesians, a special dictionary (lexicon), consisting of a list of words and their sentiment scores, was identified.

**Figure 3.**
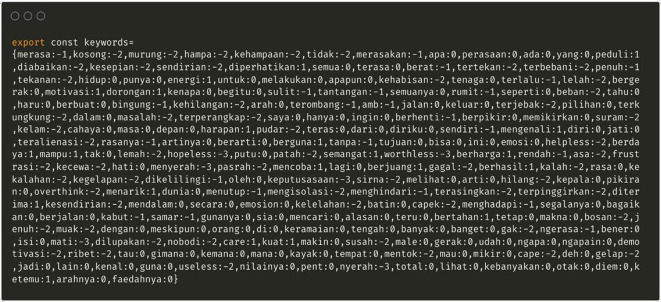
Special dictionary related to depression in Indonesian.

This list of words was based on commonly used terminology to express negative emotional states, and was obtained through discussions between research members ( Figure 3).

**Sentiment analysis process**

**Figure 4.**
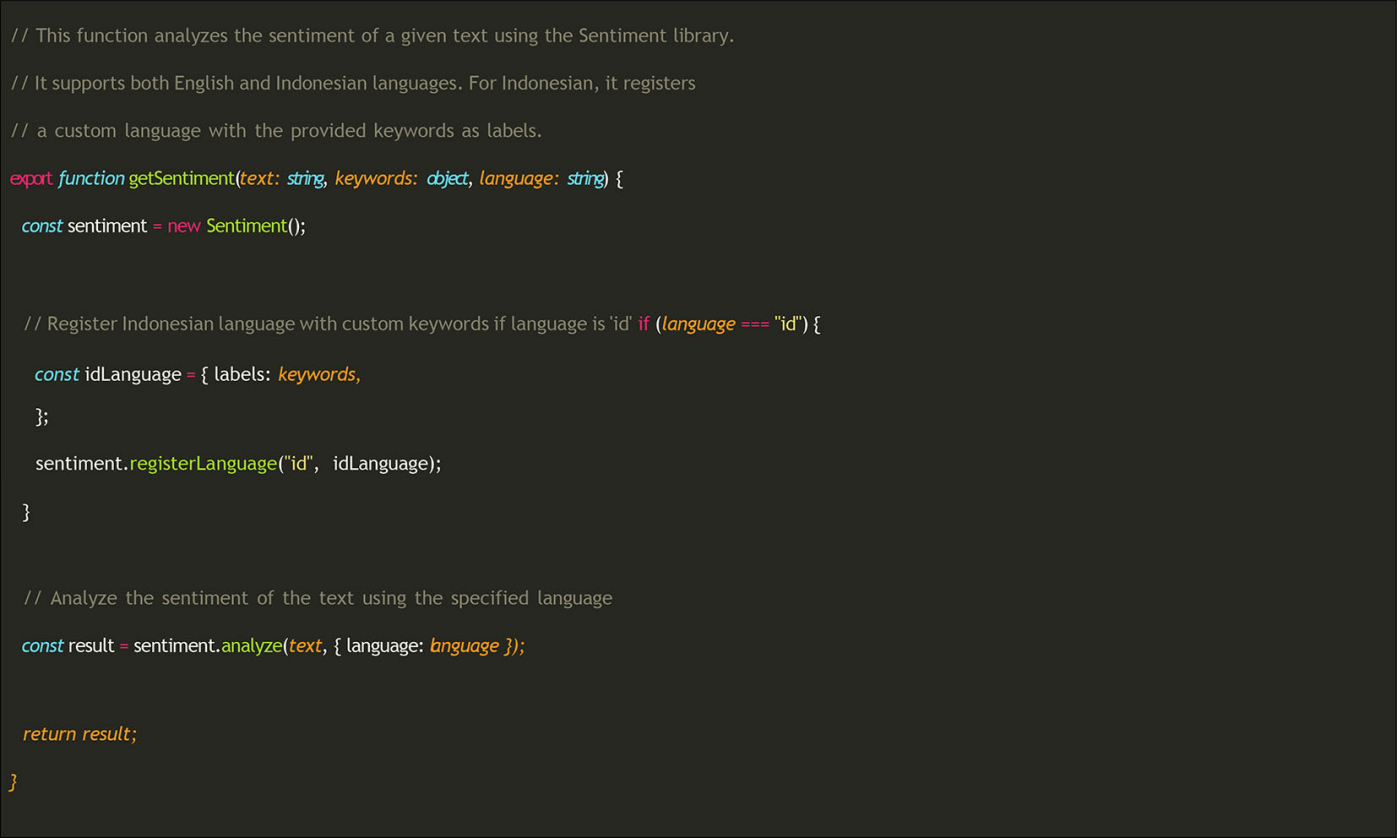
Sentiment analysis process in MOODMIND.

After the user provides voice input, which is then transcribed into text, the system performs sentiment analysis of the text. The following functions were used to perform the analysis ( Figure 4). The getSentiment function accepts three parameters: the transcribed text, the sentiment dictionary, and the language code (“id” for Indonesian or “en” for English). If the selected language was Indonesian, the library was registered using a specially compiled dictionary.

**Analysis results**

The result object returned by the analysis () function contains several attributes that provide an overview of the emotional content of the analyzed text, including the score of text sentiment (positive, negative, or neutral), comparative (the normalized score value relative to the number of tokens), tokens resulting from text segmentation, words identified as having sentiment meaning, and positive/negative words recognized in the text.

By integrating this sentiment analysis, the system automatically detects emotional indicators and provides additional data for depression-screening processes. If negative sentiments related to feelings or interests are found in the last two weeks, then it is followed by closed questions.

**B. Accuracy test**

Quantitative research was carried out with *a cross-sectional design* and aimed at testing the accuracy of MOODMIND. The research population was drug-sensitive TB patients accompanied by YARSI TB Care cadres. The inclusion criteria were patients aged 17-65 years, had undergone TB treatment for more than 1 month, and were willing to be the subject of the study. Informed Consent was carried out in writing using an electronic questionnaire. Parents or guardians would be asked for written consent (using an electronic questionnaire) for patients who are 17 years old. The samples were taken by purposive sampling in the May-July 2025.

Data collection was obtained by interview, comparing the results of detection with MOODMIND and doctor’s anamnesis. The doctor’s guide in enforcing MDD was the DSM-5. ^10^ Univariate analysis using Microsoft Excel to calculate sensitivity, specificity, *positive predictive value*, and *negative predictive value.* Single blinding was done to the doctor so that she did not know the results of detection with MOODMIND.

## 4. Results

### 4.1 Use cases

**Figure 5.**
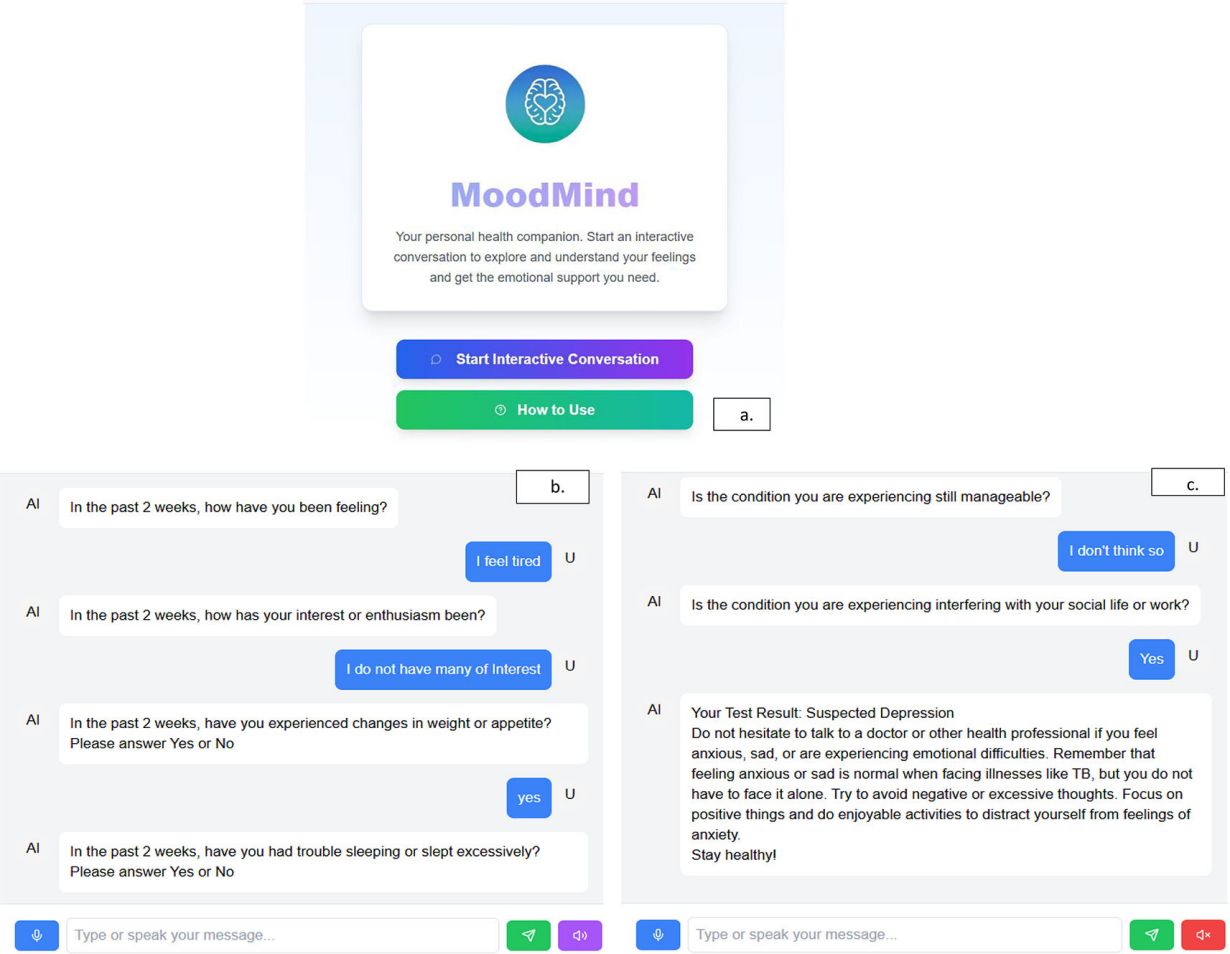
a. Front page of MOODMIND. b. Conversation flow in MOODMIND. c. Result of test in MOODMIND.

MOODMIND users can select the languages (English and Indonesian) ( Figure 5a). Users can choose either the written or voice mode of conversation ( Figure 5b). Users’ answers were categorized into 3, namely not depressed (score = 0), at risk of depression (score = 1-4), and suspected depression (score ≥ 5) ( Figure 5c). The word “Suspected depression” was used because the diagnosis by the doctor must be carried out and the patient should receive the necessary consultation. The role of a doctor/officer cannot be replaced by AI because of empathy and direct interaction with a human being.

### 4.2 Accuration test

We conducted tests on 21 patients with TB in Central Jakarta between May and July 2025. The average age of patients was 41.4 years with an age range of 19-64 years. The patient was guided by the researcher when using MOODMIND, whereas the doctor was blinded and did not know the results of the software detection.

Table 1 shows a comparison of MOODMIND detection with the doctor’s autoanamnesis, while Table 2 shows the accuracy level of the software.

**Table 1.** MOODMIND screening and doctor’s examination test results.

AI MOODMIND	Autoanamnesis		Total
	Negative	Positive	
Negative	18	1	19
Positive	0	2	2
Total	18	3	21

**Table 2.** Analysis of MOODMIND screening results on doctor’s examination.

Test	Percentage
Sensitivity	67%
Specificity	100%
Positive predictive value	100%
Negative predictive value	95%

## 5. Discussion

The MOODMIND application was equipped with sentiment analysis by searching for keywords and analyzing sentiments in Indonesian. The Lexicon technique is used to make a list of words and score sentiments for each word. ^11^ Other research has identified the keywords depression, symbols, and expressions through social media. ^12, 13^ Existing depression detection systems/applications such as “Mental Care” which asked 21 questions to respondents, ^14^ Multi-Gated LeakyReLU processed depressive language using CNN, ^15^ while another study analyzed expressions that did not directly use specific words. ^16^

Artificial intelligence usually requires the ability of the user. ^17^ However, MOODMIND is very easy to operate, which can reduce issues related to human resources. The main requirements are a device and an internet connection. This tool is an inspiration for the development of similar types in other countries according to the local language, minimizing the gap between the detected cases and the actual number of cases. The variation of words related to depression still adjusts to the current condition, so it must be continuously updated to increase sensitivity from time to time.

More sample research is needed to determine the accuracy of MOODMIND in a real-world setting. In addition, bridging the results of screening to electronic medical records can be a useful alternative for monitoring the mental health of patients with chronic diseases such as tuberculosis.

## 6. Conclusion

MOODMIND, an artificial intelligence based on Natural Language Processing, can be used as an MDD detection tool. The level of accuracy was adequate (67% sensitivity and 100% specificity). This tool supports mental health monitoring but does not replace the role of doctors. This could also be an idea for AI development in some countries to detect MDD as early as possible.

## Software availability

Source code available from: https://github.com/incrementalstudios/mood-mind

Archived software available from: https://doi.org/10.5281/zenodo.16793110 ^18^

License: MIT License

## Data Availability

The dataset as the basis for the accuracy test findings can be accessed at the link: https://doi.org/10.5281/zenodo.17114938. ^19^ We also include the approval sheets and interview guides in the link. Data are available under the terms of the Creative Commons Zero v1.0 Universal
