# Supplementary material for: MOODMIND: A Pilot Feasibility Study of Artificial Intelligence for Major Depressive Disorder Screening in Tuberculosis Patients
Source: F1000Res. 2026 Mar 19;14:1079. Originally published 2025 Oct 13. [Version 2] doi: 10.12688/f1000research.168964.2 (PMC13054254; doi:10.12688/f1000research.168964.2)
Supplement: Peer review history

# Reviewer report: Reviewer response for version 2

**DOI:** 10.5256/f1000research.197138.r468964

**Published:** 2026-04-06

**Authors:** Masab Mansoor

**Affiliations:** 1 Edward Via College of Osteopathic Medicine, Blacksburg, Virginia, USA

Thank you for your revisions. I endorse this paper for Indexing.

Are the conclusions about the tool and its performance adequately supported by the findings presented in the article?

No

Is the rationale for developing the new software tool clearly explained?

Partly

Is the description of the software tool technically sound?

No

Are sufficient details of the code, methods and analysis (if applicable) provided to allow replication of the software development and its use by others?

No

Is sufficient information provided to allow interpretation of the expected output datasets and any results generated using the tool?

Partly

Reviewer Expertise:

Machine Learning, artificial intelligence, health informatics, Large language models, Machine vision

I confirm that I have read this submission and believe that I have an appropriate level of expertise to confirm that it is of an acceptable scientific standard.

# Reviewer report: Reviewer response for version 1

**DOI:** 10.5256/f1000research.186213.r447567

**Published:** 2026-01-29

**Authors:** Hayder Al-Hindy

**Affiliations:** 1 College of Pharmacology, University of Babylon (Ringgold ID: 125654), Babylon Governorate, Babylon Governorate, Iraq

**F1000Research Software Tool Peer Review: Full Report**

 Article Summary

 This software tool article presents MOODMIND, a web-based application for screening Major Depressive Disorder (MDD) in tuberculosis (TB) patients using Natural Language Processing (NLP), specifically lexicon-based sentiment analysis on voice or text inputs in Indonesian and English. The tool poses DSM-5-derived open- and closed-ended questions, processes responses via a custom Indonesian sentiment dictionary and the JavaScript 'sentiment' library, and categorizes users as not depressed (score 0), at-risk (1-4), or suspected depression (≥5). A pilot accuracy test on 21 drug-sensitive TB patients (mean age 41.4 years) compared MOODMIND outputs to blinded physician anamnesis, yielding 67% sensitivity, 100% specificity, 100% PPV, and 95% NPV. Source code and data are openly available via GitHub and Zenodo. The manuscript highlights the tool's ease-of-use and potential for early detection in resource-limited settings but has undergone prior open peer reviews (one "approved with reservations," one "not approved").​

 Questionnaire Responses with Detailed Explanations

**1. Is the rationale for developing the new software tool clearly explained? Partly**

 The introduction establishes TB-MDD comorbidity risks, stigma, immune effects, and limitations of closed-ended questionnaires, motivating NLP for natural responses. However, it lacks quantification of MDD prevalence in Indonesian TB cohorts, explicit shortcomings of validated tools (e.g., PHQ-9's rigidity in low-literacy groups), and evidence of screening gaps this addresses.

**To address:** Add epidemiological data (e.g., local MDD-TB rates), a table comparing MOODMIND to PHQ-9/GAD-7 in TB contexts, and barriers like digital literacy or stigma. **Not essential for soundness but strengthens justification.**

**2. Is the description of the software tool technically sound? Partly**

 The architecture (Next.js, Web Speech API, custom lexicon) is outlined, with TB-specific adaptations and sentiment scoring described. However, lexicon-based analysis is outdated (vs. BERT/transformers); speech recognition lacks dialect validation; scoring logic (sentiment-to-DSM-5 symptom mapping) is opaque; neutral sentiment handling undefined.

**To address:** Provide algorithmic flowchart (input → transcription → lexicon scoring → thresholds → categorization); validate lexicon against clinical corpora with inter-rater stats; test Web Speech API accuracy on Indonesian TB dialects; specify all thresholds empirically. **Essential: Scoring/threshold details for technical validity.**

**3. Are sufficient details of the code, methods, and analysis (if applicable) provided to allow replication of the software development and its use by others? No**

 GitHub link aids access, but is missing: full get Sentiment code; lexicon weights; dependency versions/commit hash; browser/system requirements; physician interview guide; sample size/power calculation; STARD flowchart; handling of incompletes/technical failures. Purposive sampling vague (no exclusions for psychosis/substances).

**To address (all essential for soundness):**

- Supplementary file with complete lexicon (words + weights), full code snippets, version specs.
- Detailed methods: physician protocol (SCID/MINI preferred over "anamnesis"), exclusions, recruitment flowchart.
- Stats: 95% CIs, F1/AUC in tables; acknowledge n=21 pilot.
**These gaps prevent replication; must be fixed.**

**4. Is sufficient information provided to allow interpretation of the expected output datasets and any results generated using the tool? Partly**

 Figures 5a-c illustrate user flow/outputs clearly, with appropriate "suspected depression" disclaimer. However, no clinical action guidance (e.g., "at-risk" referrals); underlying scores/symptoms not shown; no rescreening protocols; false-negative risks (33% with 67% sensitivity) unquantified; suicidality absent.

**To address:** Add output interpretation guide (e.g., "at-risk → prompt PHQ-9"); symptom-level feedback; suicidality question with crisis links. Include workflow integration (e.g., TB clinic timing). **Essential: Suicidality/safety protocols to prevent harm.**

**5. Are the conclusions about the tool and its performance adequately supported by the findings presented in the article? No**

 "Adequate accuracy" overstates n=21 pilot (only 3 MDD cases; specificity CI likely 82-100%); no PHQ-9 benchmark; ignores false-negatives' TB adherence risks. Generalizability limited (supervised use, single-center).

**To address (all essential):**

- Revise to "preliminary pilot accuracy requiring validation."
- Add CIs (e.g., sensitivity 9-99%); compare to PHQ-9 (88% sensitivity).
- Explicit limitations section: small n, overfitting risk, no external cohort.
- Tone down: "Proof-of-concept for larger studies." **Overclaims undermine credibility; must be corrected.**

Strengths

 Innovative open-ended NLP for TB-MDD screening; bilingual/open-source; ethical (blinding, consent); addresses real comorbidity need in low-resource Indonesia.​

 Overall Concerns and Minor Issues

 Small sample yields unreliable metrics; outdated lexicon risks bias; safety gaps (no suicidality); typos ("Accuration," inconsistent "autoanamnesis"); vague English lexicon testing. Prior reviews echo these—manuscript needs tracked revisions.​

 Points That Must Be Addressed for Scientific Soundness

1. **Technical replication:** Full lexicon/code/thresholds (supplementary).
2. **Validation rigor:** CIs/F1; structured gold-standard (SCID); recruitment flowchart.
3. **Safety:** Suicidality screening/referrals.
4. **Tempered claims:** "Pilot" framing; PHQ-9 comparisons; limitations section.
5. **Methods clarity:** Sampling exclusions, speech validation, scoring algorithm.
  Without these, the article risks misleading clinical adoption.

Final Recommendation

**Approved with reservations.** Promising pilot but requires major revisions above for soundness. Re-review post-changes recommended.​

Are the conclusions about the tool and its performance adequately supported by the findings presented in the article?

No

Is the rationale for developing the new software tool clearly explained?

Partly

Is the description of the software tool technically sound?

Partly

Are sufficient details of the code, methods and analysis (if applicable) provided to allow replication of the software development and its use by others?

No

Is sufficient information provided to allow interpretation of the expected output datasets and any results generated using the tool?

Partly

Reviewer Expertise:

General and cardiac physiology, genetics, Pediatrics,

I confirm that I have read this submission and believe that I have an appropriate level of expertise to confirm that it is of an acceptable scientific standard, however I have significant reservations, as outlined above.

# Reviewer report: Reviewer response for version 1

**DOI:** 10.5256/f1000research.186213.r435547

**Published:** 2026-01-13

**Authors:** Caroline W. Espinola

**Affiliations:** 1 McMaster University, Hamilton, Canada

This manuscript describes an AI tool that uses natural language processing from questions derived from the DSM-5 for a screening of depression in a convenience sample of 21 patients with tuberculosis. They report an sensitivity of 67% and specificity of 100%.

 The Abstract is generic and unclear. Needs a more detailed description of the background, rationale, population studied. A clear description of the methods, including model used, is also missing. No information on the sample is provided. In the Methods, the reader would appreciate a more detailed description of the MOODMIND tool. How was NLP obtained? Which sentiment analysis techniques were used? Results are poorly reported, e.g., F1 score, AUC are not reported. Conclusion does not mention TB (the condition being studied) and makes strong assumption without justifying it. What is "Ease" in the conclusion?

 Introduction: I would add more information on the standard treatment for TB. I would also recommend adding more details, such as magnitude of risk of MDD associated with TB compared with the general population or risk of depression in people with TB receiving second and third-line therapies. Additionally, what is the impact of these changes in immune system associated with MDD on TB treatment outcomes? In the fifth paragraph, CNN and RNN are ML architetures that are used in NLP datasets, not NLP techniques by themselves. I suggest revising. 

 Methods: More information would be appreciated on the MOODMIND model and what it entails. What data was used for input - just the open-ended questions or also the other 7 closed-ended questions? Considering a diagnosis of depression requires at least 5 criteria, this could have a significant impact on the content generated, and consequently, the prediction ability of the NLP model. 

 - *3.4 User experience flow: *In the voice converted to text, what was the transcription software used -your own or a third-party one? In case of using your own, was it validated prior to this research? Where was data stored (this could be a privacy concern). Did you run any analysis on potential mistakes in the transcription software?

*3.5 Adaptation for tuberculosis:* *MOODMIND was adapted with a custom sentiment dictionary, focusing on common terms in Bahasa Indonesia that were reported by patients with TB when experiencing emotional distress. - *How are these terms different between TB and the general population. A non-Indonesian reader would appreciate more information. What about its use in English? What percentage of the sample responded in English? Was there any difference in accuracy between the English speaking and Indonesian speaking samples?

 - Figure 4: Code of the function getSentiment is not provided. Unsure of what it does. I suggest either removing this figure or adding the code for the function

 Results:

 - *4.1 Use cases: *First sentence should be moved to Methods. " *The role of a doctor/officer cannot be replaced by AI because of empathy and direct interaction with a human being." - *Not only because of these. The clinical interview is the gold standard for the diagnosis of depression. Suggest revising.

*- 4.2 Accuration test:* Since you used mean age, was the SD of age of the sample? How many patients were approached but either did not consent of were excluded. A detailed description of the eligibility criteria is needed.

 Results also need to be provided in plain text, not only in tables

 Discussion: The discussion is confusing and does not summarize the goals and results of the study. Furthermore, I understand the greatest contribution of this tool would be as a screening tool for MDD. A sensitivity of 67% is modest for screening tool, which should be able to detect true positives.This consideration should be included in the discussion. The discussion should contemplate how this tool could help detect TB patients at a higher risk for depression, which would warrant a detailed clinical assessment. Are these patients automatically flagged for the healthcare team or do they need to seek help on their own?

Are the conclusions about the tool and its performance adequately supported by the findings presented in the article?

Partly

Is the rationale for developing the new software tool clearly explained?

No

Is the description of the software tool technically sound?

Partly

Are sufficient details of the code, methods and analysis (if applicable) provided to allow replication of the software development and its use by others?

No

Is sufficient information provided to allow interpretation of the expected output datasets and any results generated using the tool?

Partly

Reviewer Expertise:

Major depressive disorder, digital mental health, brain stimulation

I confirm that I have read this submission and believe that I have an appropriate level of expertise to confirm that it is of an acceptable scientific standard, however I have significant reservations, as outlined above.

## References

[1] : Addressing the tuberculosis–depression syndemic to end the tuberculosis epidemic. *The International Journal of Tuberculosis and Lung Disease* .2017;21(8) : 10.5588/ijtld.16.0584 852-861 10.5588/ijtld.16.0584 PMC575933328786792

# Reviewer report: Reviewer response for version 1

**DOI:** 10.5256/f1000research.186213.r435555

**Published:** 2025-12-30

**Authors:** Masab Mansoor

**Affiliations:** 1 Edward Via College of Osteopathic Medicine, Blacksburg, Virginia, USA

PEER REVIEW REPORT

 SUMMARY

 This manuscript presents MOODMIND, a web-based Natural Language Processing (NLP) application designed to screen for Major Depressive Disorder (MDD) in tuberculosis patients. The tool employs lexicon-based sentiment analysis on both voice and text inputs in Indonesian and English, utilizing DSM-5 criteria. A pilot validation study with 21 TB patients demonstrated 67% sensitivity and 100% specificity compared to physician assessment.

 While the authors address a clinically relevant problem and present an accessible tool, significant methodological and technical limitations require substantial revision before indexing.

 DETAILED RESPONSES TO REVIEW QUESTIONS

 1. Is the rationale for developing the new software tool clearly explained?

**Answer: PARTLY**

**Strengths:**

- The co-morbidity between TB and MDD is well-established in the introduction
- The need for accessible screening tools is apparent
- The rationale for using open-ended questions is mentioned

**Weaknesses:**

- **Lacks justification for why existing validated tools (PHQ-9, GAD-7, BDI-II) are inadequate** for this population
- Does not explain why sentiment analysis is superior to structured questionnaires
- **Insufficient epidemiological data** on MDD prevalence specifically in Indonesian TB patients
- No discussion of barriers to current screening practices that this tool addresses
- Missing cost-effectiveness or accessibility arguments compared to existing digital mental health tools

**Recommendations:**

- Provide systematic comparison of existing MDD screening tools and their limitations in TB populations
- Clarify specific advantages of MOODMIND over validated instruments
- Include data on mental health service gaps in the target population

2. Is the description of the software tool technically sound?

**Answer: NO**

**Critical Technical Deficiencies:**

**A. Sentiment Analysis Methodology:**

- **Oversimplified approach:** Lexicon-based sentiment analysis is outdated; modern approaches utilize transformer-based models (BERT, GPT) or at minimum, more sophisticated machine learning classifiers
- **No description of sentiment score thresholds** for categorization into non-depressed/at-risk/suspected depression
- **Lack of validation** for the custom Indonesian lexicon (Figure 3 shows word list but no validation process)
- **No explanation of how sentiment scores correlate with DSM-5 diagnostic criteria** for MDD

**B. Algorithm Description:**

- Figure 4 shows code snippet but **insufficient detail** for replication:
  - What constitutes a "negative sentiment" threshold?
  - How are comparative scores normalized?
  - What happens with neutral sentiments?
- **Missing information on decision tree logic** for transitioning from open to closed questions
- No description of how the algorithm **weighs different DSM-5 symptoms**

**C. Speech Recognition:**

- Uses Web Speech API but **no validation of transcription accuracy** for Indonesian language
- No discussion of handling **dialectal variations** or accented speech
- **Error handling procedures** for misrecognition not addressed

**D. Scoring System:**

- **Critical gap:** The manuscript states results are categorized as score 0, 1-4, or ≥5, but never explains:
  - How these numeric scores are generated from sentiment analysis
  - What each point represents (symptom count? Severity weighting?)
  - How DSM-5's requirement of "5 or more symptoms with at least 1 core symptom" maps to the scoring

**Recommendations:**

1. Provide detailed algorithmic flowchart from input to classification
2. Specify all threshold values and their empirical basis
3. Consider upgrading to modern NLP architectures
4. Validate Indonesian lexicon against clinical datasets
5. Provide comprehensive technical documentation in supplementary materials

3. Are sufficient details provided for replication?

**Answer: NO**

**Missing Critical Information:**

**A. Lexicon Development:**

- Figure 3 shows word list but:
  - **No systematic methodology** for term selection
  - "Obtained through discussions between research members" is insufficient
  - **No validation** against clinical depression corpora
  - **Sentiment weights/scores** for each term not provided
  - No inter-rater agreement statistics for lexicon development

**B. Software Implementation:**

- While GitHub repository is referenced:
  - **Version control information** missing (which commit/release tested?)
  - **Dependency versions** not specified
  - **Deployment environment** specifications absent
  - Browser compatibility not documented

**C. Validation Protocol:**

- **Insufficient detail on physician assessment:**
  - What specific questions did physicians ask?
  - How long were clinical interviews?
  - What documentation was completed?
  - Single physician or multiple (inter-rater reliability)?
- **Sampling procedure:** "Purposive sampling" requires more specificity
- **Inclusion/exclusion criteria** need expansion (e.g., cognitive impairment, substance use, psychotic disorders)

**D. Statistical Analysis:**

- **No sample size calculation or power analysis**
- **Confidence intervals** not provided for sensitivity/specificity
- **Missing information on handling:**
  - Incomplete responses
  - Technical failures
  - Participant withdrawals

**Recommendations:**

1. Publish complete lexicon with sentiment weights as supplementary data
2. Provide structured interview guide used by physicians
3. Include detailed statistical analysis plan
4. Add flowchart of participant recruitment and assessment
5. Specify all software versions and system requirements

4. Is sufficient information provided to interpret expected outputs?

**Answer: PARTLY**

**Strengths:**

- Figure 5c shows example output interface
- Three-category classification (not depressed/at-risk/suspected) is clear
- Appropriate disclaimer about need for professional diagnosis

**Weaknesses:**

**A. Output Interpretation:**

- **No guidance on clinical action** for each category:
  - What should clinicians do with "at-risk" patients?
  - Referral pathways not discussed
  - Urgency assessment absent
- **Score explanation missing:** Users see categorical result but not underlying score or contributing factors
- **No feedback on specific symptoms** identified

**B. Clinical Utility:**

- **Unclear integration with clinical workflow:**
  - When in TB treatment should screening occur?
  - How often should rescreening happen?
  - Documentation recommendations?
- **False negative implications** not discussed (with 67% sensitivity, 33% of MDD cases missed)

**C. Limitations Communication:**

- Tool appropriately states it doesn't replace clinical diagnosis, but:
  - **Doesn't explain to users why** (especially for low-literacy populations)
  - **Risk of delayed care** if patients with negative screens don't seek help
  - **Suicidality assessment** completely absent

**Recommendations:**

1. Provide detailed clinical implementation guidelines
2. Include symptom-level feedback in output
3. Add explicit suicidality screening and immediate referral protocols
4. Create user education materials explaining tool limitations
5. Develop clinician interpretation guide with case examples

5. Are conclusions adequately supported by findings?

**Answer: NO**

**Major Concerns:**

**A. Overstated Claims:**

- **"Adequate accuracy" (67% sensitivity)** is debatable for screening tool:
  - Missing 1 in 3 cases is problematic for MDD screening
  - Compare to PHQ-9: sensitivity ~88%, specificity ~88% for MDD diagnosis
  - **No justification** for why 67% is acceptable
- **"100% specificity"** is misleading:
  - Based on **zero false positives in sample of 18 true negatives**
  - With 95% confidence interval, true specificity could be as low as ~82%
  - Overfitting likely with such small sample

**B. Statistical Limitations:**

- **Sample size (n=21) severely underpowered:**
  - Only 3 MDD-positive cases
  - Cannot reliably estimate diagnostic accuracy
  - No subgroup analyses possible
  - Results not generalizable
- **Selection bias:** Patients "accompanied by YARSI TB Care cadres" may not represent broader TB population
- **Verification bias:** Single physician assessment without structured interview or validated scales

**C. Comparative Evidence:**

- **No comparison with validated screening tools** in same population
- **No benchmark** against PHQ-9, which is:
  - Free, brief (9 items)
  - Extensively validated in medical populations
  - Available in Indonesian

**D. Generalizability:**

- **Single-center study** in Central Jakarta
- **TB patients only** - MDD presentation may differ in other chronic diseases
- **Researcher-supervised administration** - real-world performance likely lower
- **Indonesian language validation** insufficient (no dialectal testing)

**E. Missing Discussions:**

- **False negative consequences:** Untreated depression worsens TB outcomes, adherence
- **Screening frequency:** Optimal timing during 6-month TB treatment unclear
- **Cost-effectiveness:** Not analyzed
- **Implementation barriers:** Internet access, device availability, digital literacy

**Recommendations:**

1. Revise conclusions to acknowledge substantial limitations
2. Clearly state this is a **proof-of-concept pilot** requiring extensive validation
3. Compare performance to PHQ-9 in future studies
4. Conduct multi-center validation with ≥300 participants
5. Include external validation cohort
6. Perform head-to-head comparison with validated instruments

ADDITIONAL MAJOR CONCERNS

 Methodological Issues:

1. **Gold Standard Inadequate:**
  - Physician "autoanamnesis" is not validated
  - Should use Structured Clinical Interview for DSM-5 (SCID) or MINI
  - Consider including validated self-report measures as convergent validity
2. **Blinding Incomplete:**
  - Researchers present during MOODMIND administration could influence responses
  - Should be independently administered without researcher presence
3. **Missing Data on:**
  - TB disease characteristics (drug-sensitive only stated, but severity, treatment phase?)
  - Psychiatric history (first episode vs. recurrent MDD?)
  - Current psychotropic medications
  - Comorbid psychiatric conditions
  - Sociodemographic factors

Ethical Concerns:

1. **Vulnerable Population:**
  - TB patients with MDD are doubly stigmatized
  - Data security measures not described
  - Privacy protections for voice recordings unclear
2. **Suicidality Risk:**
  - **No suicide risk assessment** in the tool
  - **Critical safety gap:** MDD screening without suicide screening is dangerous
  - **No crisis referral pathway** described

Technical Concerns:

1. **Open-Ended Question Analysis:**
  - How are diverse, unstructured responses converted to binary symptom presence/absence?
  - Inter-rater reliability for human coding not established
  - Automated coding validation absent
2. **Closed Question Integration:**
  - Manuscript states both open and closed questions used
  - **Logic for triggering closed questions** not explained
  - **Scoring methodology** for combined responses unclear

SPECIFIC CORRECTIONS REQUIRED

 Methods Section:

1. **Line describing sample:** Change "21 patients" to "21 patients (pilot feasibility study)"
2. **Add:** Structured clinical interview protocol used by physician
3. **Add:** Sample size justification or acknowledge as convenience sample
4. **Add:** Inter-rater reliability assessment (if multiple raters) or acknowledge single-rater limitation

Results Section:

1. **Add:** 95% confidence intervals for all diagnostic accuracy metrics
2. **Add:** Participant flowchart (STARD guidelines)
3. **Add:** Description of any technical failures or incomplete assessments
4. **Modify Table 2:** Include confidence intervals

Discussion Section:

1. **Add:** Direct comparison of 67% sensitivity to literature values for validated tools
2. **Add:** Clinical implications of 33% false negative rate
3. **Add:** Limitations section discussing:
  - Small sample size and wide confidence intervals
  - Lack of external validation
  - Single-center, single-assessor design
  - Absence of comparison to validated instruments
4. **Add:** Implementation research needs before clinical deployment

Conclusion Section:

1. **Modify:** Change "adequate accuracy" to "preliminary accuracy estimates requiring validation in larger studies"
2. **Add:** Explicit statement: "This tool requires extensive validation before clinical implementation"
3. **Add:** Specific next steps for validation research

MINOR ISSUES

 Writing Quality:

- Generally clear but some grammatical errors
- "Autoanamnesis" - unusual term; clarify or use "clinical interview"
- Inconsistent terminology (MDD vs. depression vs. major depression)

Figures:

- Figure 1: Simplistic - could be removed or enhanced with algorithm specifics
- Figure 2: Helpful conceptual framework
- Figure 3: Shows code but insufficient explanation
- Figure 4: Code snippet needs more context
- Figure 5: Good interface examples but needs annotation

References:

- Appropriate selection
- Missing key references on digital mental health tools
- Should cite PHQ-9 validation studies in TB populations (if available)

VERDICT AND RECOMMENDATIONS

 Overall Assessment: **MAJOR REVISIONS REQUIRED**

**Must Be Addressed for Scientific Soundness:**

1. **Acknowledge severe limitations** of 21-patient pilot study throughout manuscript
2. **Revise conclusions** to reflect preliminary nature of findings
3. **Add confidence intervals** to all diagnostic accuracy estimates
4. **Provide complete technical documentation** sufficient for replication
5. **Include suicide risk assessment** in tool or acknowledge dangerous omission
6. **Explain scoring algorithm** in detail
7. **Validate lexicon** using established methodology
8. **Compare to validated screening tools** (add as limitation if not done)

**Strongly Recommended:**

1. Conduct adequately powered validation study (n≥300) before claiming clinical utility
2. Perform external validation in different TB treatment settings
3. Include structured clinical interview as gold standard
4. Assess inter-rater reliability
5. Upgrade NLP methodology to modern standards
6. Publish complete source code and lexicon with version control

Suggested Title Revision:

 "MOODMIND: A Pilot Feasibility Study of Artificial Intelligence for Major Depressive Disorder Screening in Tuberculosis Patients"

 Alternative Publication Path:

 Given the early-stage development and small sample, authors might consider:

- Repositioning as a "Software Tool Note" rather than validation study
- Focus on technical description with clear acknowledgment that clinical validation is pending
- Present diagnostic accuracy data as preliminary feasibility only

CONCLUSION

 While MOODMIND addresses an important clinical need and demonstrates creative application of NLP to mental health screening, the manuscript requires substantial revision to meet scientific standards for a clinical validation study. The combination of outdated NLP methodology, inadequate technical description, severely underpowered validation study, and absence of critical safety features (suicide screening) precludes recommendation for approval in current form.

 The authors should be commended for open-source development and bilingual implementation, but must conduct rigorous validation research before clinical deployment recommendations can be supported.

**Recommendation: MAJOR REVISIONS REQUIRED before this manuscript can be considered for Indexing.**

Are the conclusions about the tool and its performance adequately supported by the findings presented in the article?

No

Is the rationale for developing the new software tool clearly explained?

Partly

Is the description of the software tool technically sound?

No

Are sufficient details of the code, methods and analysis (if applicable) provided to allow replication of the software development and its use by others?

No

Is sufficient information provided to allow interpretation of the expected output datasets and any results generated using the tool?

Partly

Reviewer Expertise:

Machine Learning, artificial intelligence, health informatics, Large language models, Machine vision

I confirm that I have read this submission and believe that I have an appropriate level of expertise to state that I do not consider it to be of an acceptable scientific standard, for reasons outlined above.
